# Effectiveness of chiropractic manipulation versus sham manipulation for recurrent headaches in children aged 7–14 years - a randomised clinical trial

**DOI:** 10.1186/s12998-020-00360-3

**Published:** 2021-01-07

**Authors:** Susanne Lynge, Kristina Boe Dissing, Werner Vach, Henrik Wulff Christensen, Lise Hestbaek

**Affiliations:** 1 Private Chiropractic Practice, Vivaldisvej 6, 9700 Broenderslev, Denmark; 2 Department of Sports Science and Clinical Biomechanics, University of Southern Denmark, Campusvej 55, 5230 Odense M, Denmark; 3 Nordic Institute for Chiropractic and Clinical Biomechanics, Campusvej 55, 5230 Odense M, Denmark; 4 Basel Academy, Steinenring 6, 4054 Basel, Switzerland; 5 Private Chiropractic Practice, Enghavevej 2, 5800 Nyborg, Denmark

**Keywords:** Ηeadache, Chiropractic, Children, Manipulation, Adolescents, Effectiveness, Clinical trial

## Abstract

**Background:**

To investigate the effectiveness of chiropractic spinal manipulation versus sham manipulation in children aged 7–14 with recurrent headaches.

**Methods:**

*Design*: A two-arm, single-blind, superiority randomised controlled trial.

*Setting*: One chiropractic clinic and one paediatric specialty practice in Denmark, November 2015 to August 2020.

*Participants*: 199 children aged 7 to 14 years, with at least one episode of headache per week for the previous 6 months and at least one musculoskeletal dysfunction identified.

*Interventions*: All participants received standard oral and written advice to reduce headaches. In addition, children in the active treatment group received chiropractic spinal manipulation and children in the control group received sham manipulation for a period of 4 months. Number and frequency of treatments were based on the chiropractor’s individual evaluation in the active treatment group; the children in the control group received approximately eight visits during the treatment period.

*Primary outcome measures:* ‘Number of days with headache’, ‘pain intensity’ and ‘medication’ were reported weekly by text messages, and global perceived effect by text message after 4 months. A planned fixed sequence strategy based on an initial outcome data analysis was used to prioritize outcomes. ‘Number of days with headache’ and ‘pain intensity’ were chosen as equally important outcomes of highest priority, followed by global perceived effect and medication. The significance level for the first two outcomes was fixed to 0.025 to take multiplicity into account.

**Results:**

Chiropractic spinal manipulation resulted in significantly fewer days with headaches (reduction of 0.81 vs. 0.41, *p* = 0.019, NNT = 7 for 20% improvement) and better global perceived effect (dichotomized into improved/not improved, OR = 2.8 (95% CI: 1.5–5.3), NNT = 5) compared with a sham manipulation procedure. There was no difference between groups for pain intensity during headache episodes. Due to methodological shortcomings, no conclusions could be drawn about medication use.

**Conclusions:**

Chiropractic spinal manipulation resulted in fewer headaches and higher global perceived effect, with only minor side effects. It did not lower the intensity of the headaches.

Since the treatment is easily applicable, of low cost and minor side effects, chiropractic spinal manipulation might be considered as a valuable treatment option for children with recurrent headaches.

**Trial registration:**

ClinicalTrials.gov, identifier NCT02684916, registered 02/18/2016 – retrospectively registered.

**Supplementary Information:**

The online version contains supplementary material available at 10.1186/s12998-020-00360-3.

## Background

Recurrent paediatric headache is common with annual prevalence rates ranging from approximately 5% among 3-year-olds to more than half of the population around puberty [1]. Recurrent headaches affect quality of life in children and are known to interfere with school performance [2, 3], social life among peers and family [2], and participation in play and sports [4]. Children with recurrent headaches also report higher levels of stress and depression compared with children without headache [3]. Importantly, suffering from recurrent childhood headache can be a precursor to potentially severe headache syndromes later in life [5].

Paediatric headaches can be complex and are often associated with co-morbid conditions [6, 7] and the aetiology is diverse, including familial disposition [8], psychological factors [9, 10], nutrition [11], socioeconomic factors [7, 12] and more. Trauma to the head or neck, as well as prolonged static postures, have also been associated with headache in children [13–15]. Thus, a biomechanical element may be involved in the aetiology and therefore, spinal manipulation has been suggested as a treatment for headaches. There is some evidence for the effectiveness of spinal manipulation in adults with chronic headache [16, 17]. Spinal manipulation is not uncommon for children with headache, as it is the presenting symptom for 11–20% of school-aged children and adolescents in chiropractic practice [18–20]. Nevertheless, with the exception of one small study where the children only received a single manipulative treatment [21], which is not common practice [16, 22], there has not been any formal investigation into the effectiveness of spinal manipulation for children with recurrent headache.

Given the potentially severe consequences of recurrent headaches in childhood and the risk of lifelong trajectories of pain, safe and effective management needs to be identified. Adverse events following spinal manipulation appear to be very rare [23] and the risk to be less than after taking medication, which is often prescribed for painful conditions [24]. Therefore, this approach calls for investigation.

## Methods

### Aim

The aim of this study is to investigate the effectiveness of chiropractic spinal manipulation versus sham manipulation in children aged 7–14 with recurrent headaches.

### Trial design

This was a two-arm, single-blind, superiority randomized controlled trial. The protocol of the study has been published elsewhere [25].

### Participants

Between November 2015 and August 2019, we recruited participants for this trial through the Danish School Information Network, local newspapers, television, social media and radio. The children had to be 7–14 years old, to have experienced at least one episode of headache per week for the previous 6 months and to have at least one musculoskeletal dysfunction in the spine, pelvis and/or temporomandibular joint, identified by the investigating chiropractor. Exclusion criteria were examination findings requiring immediate referral, contraindications to spinal manipulation, previous treatment for headache within the last 3 months and failure to report pre-randomisation baseline data.

### Setting

The study took place at two clinics in Northern Denmark: one chiropractic clinic and one paediatric specialty practice. Screenings and treatments were administered in both clinics by the same investigating chiropractor with 34 years of experience in private practice.

### Pre-randomisation data collection

Before screening, eligible children and their parents answered three questions each Sunday for 4 weeks via a text message on their cell phone (Short Message System, SMS). The questions regarded number of days with a headache, intensity of headaches and number of pills taken for headache during the previous week. In addition, a pre-treatment questionnaire including the characteristics of the child’s headache problem, lifestyle, previous trauma, previous treatment, family history of headache and general health was completed and returned by mail. Details have been reported in the protocol [25].

### Screening

After a four-week pre-treatment period, verifying at least four episodes of headache, a screening for inclusion/exclusion was done by the investigating chiropractor. This included standard neurologic and orthopaedic examination, as well as examination for biomechanical dysfunctions in the spine, pelvis and temporomandibular joints.

### Randomisation

Upon receipt of the signed consent form, participating children were randomised with 1:1 allocation using random block size with the software nQuery Advisor [26] by the data manager at the Nordic Institute for Chiropractic and Clinical Biomechanics. Group assignment was noted in opaque envelopes and sent to the project clinics.

After completing the four-week pre-treatment period and confirmed as eligible for inclusion at the screening visit, all participants and their parents received oral and written advice believed to be beneficial to headache patients in general, regarding regular sleep, diet and exercise. After this the randomisation envelope was opened by the investigating chiropractor and the trial period of 4 months began.

### Intervention

At all the visits, the participants’ parents were present during a short interview where information about side effects and trauma experienced since the previous visit was collected by the chiropractor. The parents would then leave the room, and after examining the child, either the chiropractic spinal manipulation or the sham manipulation was administered.

The chiropractic spinal manipulation treatment was directed at specific, individually identified dysfunctions of one or more joints in the spine, pelvis and/or temporomandibular joints. A high-velocity, low-amplitude thrust, resulting in an audible cavitation, was given to improve the function of the joint. The treatment has been described in detail in the study protocol [25]. All treatments were modified to fit the age and size of the child as well as individual spinal characteristics. To reflect daily clinical practice, the number and frequency of treatments, as well as the joints treated, were based on the chiropractor’s individual evaluation at each visit throughout the 4 months treatment period.

The sham manipulation treatment followed a previously validated protocol, developed by Chaibi et al. [27]. Placement of the child was similar to the placement in the chiropractic spinal manipulation group, but low-amplitude, low-velocity gentle pushes in a broad non-specific contact away from the spinal column were given with no resulting cavitation. In addition to the protocol previously established by Chaibi et al., a de-activated activator (www.activator.com) [28] on the chiropractor’s own arm would produce a click-noise in connection with the cervical treatment to resemble the sound of the audible joint cavitation in the chiropractic spinal manipulation group. The children in this group should receive approximately eight visits with increasing intervals during the 4 months participation period to resemble a common course of care in a chiropractic practice. The ideal schedule was 2 visits the first week, 1 visit/week the following 2 weeks, 1 visit every other week for 4 weeks and finally the last two visits 4 weeks apart, but it could be modified to the parents’ convenience.

### Post-intervention treatment

Children in the chiropractic spinal manipulation treatment group who reported little or no effect, or a worsening of their headache after treatment were offered a consultation with the paediatrician. Children in the sham manipulation group who reported little or no effect, or a worsening of headache after the trial period were offered free chiropractic care, similar to the care delivered in the spinal manipulation group. After the four-month post-trial treatment period, parents received a final text message, identical to the one they received after participating in the trial regarding the effect of the treatment.

### Outcomes

Throughout the study period, the parents together with their participating children answered the same weekly text messages as they had during the pre-randomisation period:

1. “How many days has <child’s name> had a headache this week? Choose a number between 0 and 7”.
2. “How will you rate the pain on a scale from 0-10, where 0 is no pain and 10 is the worst pain you can imagine?”
3. “How many pills for headache has < child’s name> taken this week? 0: none, 1: 1-4, 2: more than 4 pills.”

The parents sent the answers using the reply function, and the answers were automatically registered and stored in a database. At the end of the 4 months of treatment, all participating families received a final text message including three questions to be answered in collaboration between parents and child:

1. “How satisfied is <child’s name> with participation in this trial on a scale from 0-10, where 0 is the worst and 10 is the best you can imagine?”
2. “How has the headache changed since <child’s name> started the treatment at the chiropractor? 1. almost gone/disappeared; 2. much better; 3. slightly better; 4. same; 5. a little worse; 6. much worse; 7. worse than ever.”
3. “In this trial there have been two groups. Do you think that <child’s name< was in group 1, who had standard chiropractic treatment or in group 2, that DID NOT have standard chiropractic treatment (please answer 1 or 2)?”

To estimate the effect of the intervention, we considered the average values during the pre-treatment period and the final 4 weeks of the study period (Weeks 14–17) for the three variables based on the weekly SMS questions. The final outcomes were then given by the change scores, i.e. the difference between these average values. A fourth outcome was the global perceived effect (GPE) based on the SMS (question #2) after 4 months.

### Sample size

A sample size calculation was conducted when the data collection was completed for 50 children in each treatment group [29]. This was based on weekly headache days, and details were reported in the study protocol [25]. A sample size of 100 children in each group was indicated to detect a difference of 20% in mean change score between groups with a power of 80% and a significance level of 5%. Calculations were performed in nQuery Advisor [26]. Allowing for a 20% drop-out rate, the aim for inclusion was 240 children. However, as drop out was very rare, inclusion was terminated after 199 children.

### Blinding

Blinding the chiropractor was obviously not possible. Allocation was concealed from the participants and their parents and blinding was further attempted by including a sham manipulation, closely resembling the active treatment, including the clicking sound. At the end of the treatment period, participating children and their parents received a text message asking which group they believed the child had participated in.

### Initial outcome analysis

Due to a lack of experience with the four potential outcomes in a population of children suffering from headache and with an SMS-based data collection based on responses from children and their parents, we did not know whether they were measured in a reliable manner and whether they would show a population variation suitable to be used as an outcome in an RCT. For example, we could not exclude that there would be little variation in some of the intended outcomes across children, or that we observed associations with baseline variables which were lower than expected and/or difficult to explain. Such insights were needed to make an informed prioritisation of the outcome variables. Therefore, we conducted an interim outcome data analysis to avoid potential misjudgments [30, 31].

That interim analysis was performed blinded to intervention status to provide information about the distribution of the four outcome variables in our population and guide the final prioritisation of outcome measures. The results of this analysis were discussed among the authors and the resulting decision report was approved by all authors before analyses of effects were initiated. The statistical report and the final decision report can be found in the Additional File 1.

The following main conclusions were drawn in the report. Since some participants did not follow the instructions to cluster the number of medications in their SMS responses and report the actual number, data on medication use could only be analysed in a reliable manner by identifying the presence/absence of use each week. Consequently, the corresponding primary outcome variable was now the change in the proportion of weeks with medication use. As many children did not report any intake of medication in the pre-treatment phase, the statistical report also suggested a sensitivity analysis, which only included children with at least 2 weeks with medication in the pre-treatment phase.

The vast majority of participants reported a pain intensity of 0 in the weeks with no days of headache, in line with our expectation. However, it turned out that this led to a high variation in pain intensity over time for many children. Hence, the definition of the four-week pain scores was changed for intensity to take only weeks with at least 1 day of headache into account. Rather than illustrating the average intensity, this reflects the intensity of the headaches *when present*. In addition, two further intensity definitions were suggested to be included in the sensitivity analyses: the original definition and the average over the last available 4 weeks with headache, but maximally going back 7 weeks from the end of the treatment period. Lastly, the statistical report suggested the inclusion of an analysis of lower percentiles of the change scores, instead of means, as a sensitivity analysis to cover the scenario of only a few children benefiting from the intervention.

Based on the above, ‘number of days with headache’ and ‘pain intensity’ were chosen as equally important outcomes of highest priority, followed by GPE and then medication. The significance level for the first two outcomes was fixed to 0.025 to take multiplicity into account within the planned fixed sequence strategy.

### Statistical analyses

All analyses were performed and presented to all authors for interpretation blinded for intervention status and two alternative conclusions were formulated before the concealment was broken. Baseline characteristics are reported as frequencies in each treatment arm for binary and categorical variables, and as means for continuous variables. To illustrate the spread of the data, means are supplemented with 10th and 90th percentiles. With respect to previous examinations, previous treatments and reasons for school absence due to illness, the frequencies for the most common category are reported.

The distribution of the primary outcomes in each treatment group is illustrated by dot plots. Intervention effects are assessed by the difference in mean values between the two intervention groups. They are supplemented by t-test-based 95% confidence intervals, *p*-values and Cohen’s d (standardized mean difference between groups) as a measure of effect size. In addition, we present adjusted p-values based on a linear regression model with one covariate in addition to treatment. That covariate was the baseline level for each of the change scores, and the number of days with headache at baseline for GPE. According to the protocol, an adjustment for additional baseline characteristics was planned in case they showed a correlation of at least 0.3 with the outcome variable. However, no such characteristic was identified. The multiplicity implied by considering four primary outcomes was taken into account by applying a fixed sequence strategy according to the prioritisation resulting from the initial outcome data analyses. Significance levels of 0.025 for the first two primary outcomes and 0.05 for the two remaining were used and applied to the adjusted *p*-values.

For the three change scores, a responder analysis was conducted, reporting the proportion in each group with 20, 25, 50 and 75% improvement compared with baseline. The number needed to treat (NNT) to reach a 20% improvement is also reported. In order to decide whether to refer the child to a paediatrician after the follow-up period, GPE was dichotomised into ‘improved’ or ‘same or worse’ [25]. The same dichotomisation was used to calculate NNT and this also allowed calculation of an odds ratio.

Side effects and satisfaction with care are analysed as secondary outomes (the latter not mentioned in the study protocol). In addition, course of treatment and the parents’ guess about treatment group are reported by group. Results for the pre-specified secondary outcomes, headache status and GPE after 8 and 12 months, will be presented in a subsequent manuscript.

Aligned with the protocol, sensitivity analyses were performed with missing values imputed based on multiple imputation (described in detail in Additional File 2). In addition, the sensitivity analyses according to the initial outcome data analysis report were performed.

The study is reported according to the CONSORT guidelines [32] and a CONSORT check list is included as Additional File 3.

STATA v.16.0 (StataCorp, College Station, Tx, USA) was used for all analyses.

### Ethics

All parents were required to give written informed consent allowing their child to participate in this study and they were informed orally and in writing that participation in the trial was voluntary and that parents could withdraw their child from the trial at any time with no negative consequences for the child. All participants were treated according to the Declaration of Helsinki [33].

The project was approved by the Regional Committee on Health Research Ethics for The North Denmark Region (#N-20150025) and data were handled according to the General Data Protection Regulations [34]. The trial was registered with ClinicalTrials.gov (Identifier: NCT02684916) [35].

## Results

### Recruitment and dropouts

**Fig. 1:**
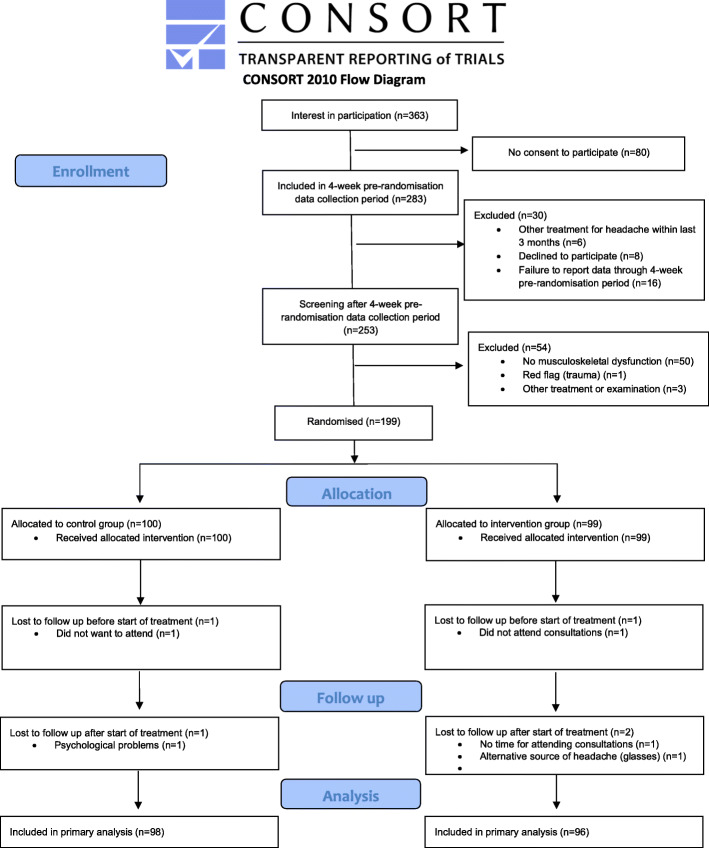
Consort flowchart

The inclusion period lasted from November 1st 2015 to September 2nd 2019. Of the 253 children screened, 199 (79%) children were eligible for inclusion. Of these, 99 were randomised to the intervention group, and 100 to the control group. Five children dropped out during the four-month trial period for reasons unrelated to treatment (Fig. 1).

In some children, the weekly SMS data were missing for some weeks or single items were missing. Hence the averages used to compute change scores were based on less than 4 weeks for some individuals. Details are given in Additional File 4.

### Patient characteristics at baseline

There were more girls (57%) than boys in the cohort and the mean age at baseline was 10.8 years. The mean pain intensity at baseline was 6.0 and the vast majority of children had taken non-prescriptive medicine at some point, most often 1–3 times per month, but only 4% had taken prescriptive medicine. Approximately half of the children had been seen by a general practitioner for their headache, but only one-fifth had received treatment for their headache, most often by a chiropractor. Approximately three-quarters of the children reported previous trauma to the head and/or neck, and headache was by far the primary reason for absence from school due to illness. The majority of the children were physically active during leisure time and had a sufficient amount of sleep. Baseline distribution of age, sex and the outcomes are shown in Table 1 and further details are presented in Additional File 5, Supplementary Table 1.

**Table 1:** Baseline values of sex, age and outcome measures. Additional baseline characteristics are shown in Additional File 5, Supplementary Table 1

	Intervention group (***N*** = 99)	Control group (***N*** = 100)
Sex, boys, n (%)	45 (45%)	40 (40%)
Age (mean, SD)	10.9 (2.1)	10.7 (2.0)
Number of days/week^a^ (mean, SD)	2.8 (1.4)	2.8 (1.5)
Pain intensity^a^ (NRS) (mean, SD)	5.2 (1.4)	5.3 (1.4)
Medication^b^ (mean, SD)	0.5 (0.3)	0.4 (0.4)

^a^within a 4-week period at baseline, ^b^proportion of weeks with medication use

### Headache characteristics at baseline

Approximately half the children had experienced recurrent headaches for 1 to 3 years, most often a few days per week, but 13% suffered from headache almost every day. An episode of headache lasted typically from 2 to 12 h per day and the onset varied over day and night. The typical region of onset varied across children, but headache was often located at the forehead. The most predominant co-occurring symptoms were nausea, dizziness, light and sound sensitivity (53–61%). Neck pain (48%) and the use of computer/tv (51%) were the activities most often thought to cause headache, whereas sleeping was the main activity easing the headache (83%). Further details are presented in Additional File 5, Supplementary Table 2.

### Analysis of primary outcomes

**Fig. 2:**
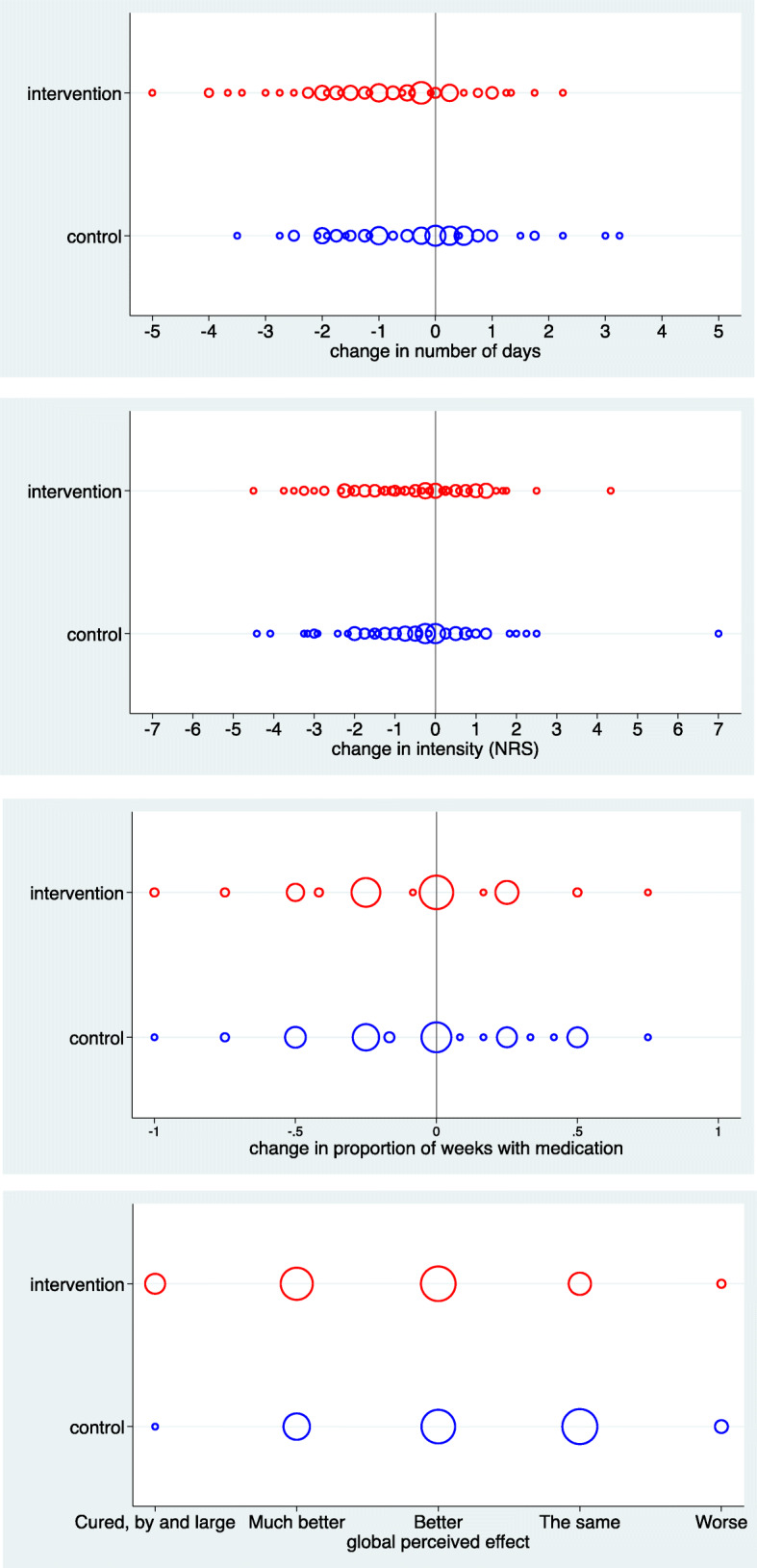
Change in the four primary outcomes by treatment group. The size of the symbols is proportional to the number of children

**Fig. 3:**
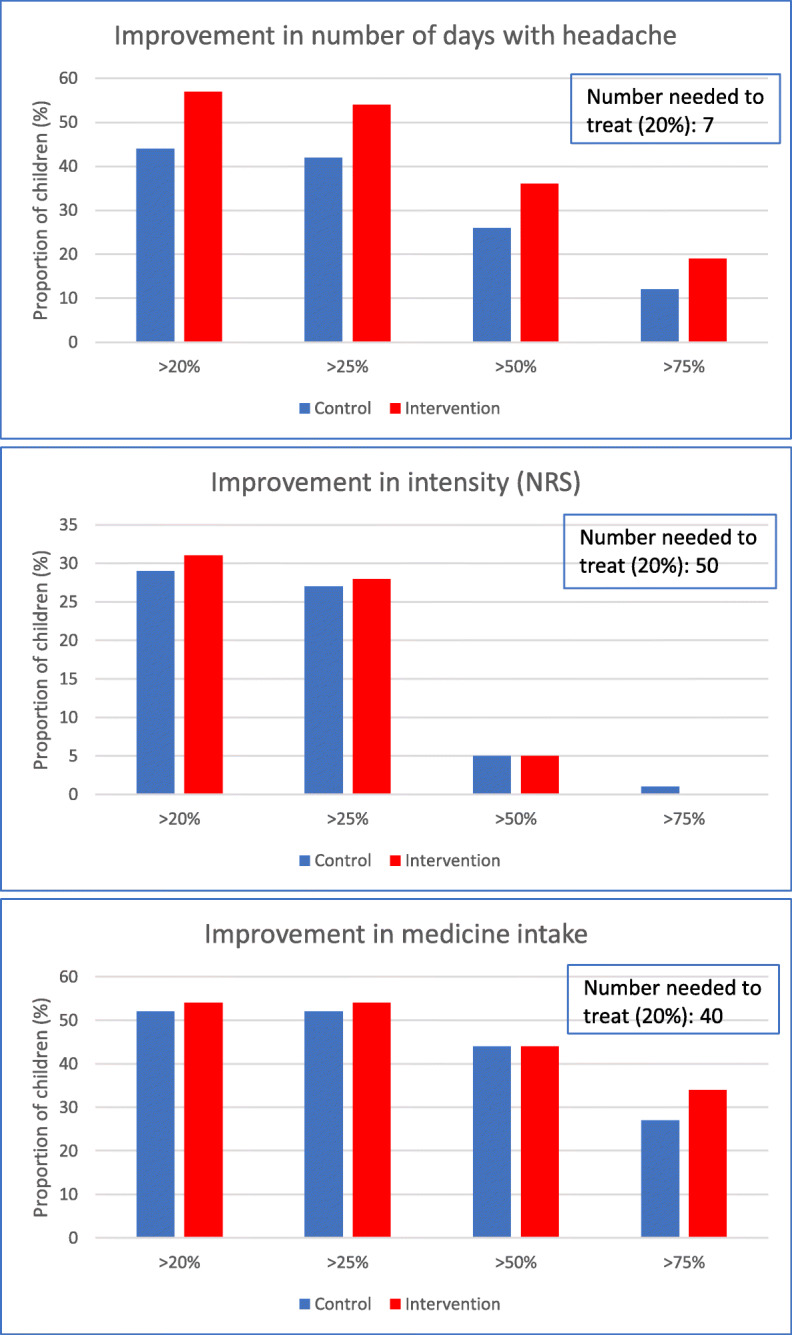
Responder analysis, including NNT, for the three continuous outcomes

Table 2 and Figs. 2 and 3 describe the results for the primary outcomes.

**Table 2:** Results on the four primary outcomes

	Control		Intervention					
Outcome	N	Mean	N	Mean	Difference in mean (95% CI)	*p*-value	*p*-value**	Cohen’s d***
Number of days per week*	97	− 0.41	96	− 0.81	−0.40 (− 0.77; − 0.05)	0.027	0.019	0.32
Intensity (NRS)*	93	−0.53	90	−0.52	0.01 (−0.43; 0.46)	0.958	0.930	0.01
GPE	98	3.24	96	2.63	−0.61 (−0.88; − 0.36)	< 0.001	< 0.001	0.67
Medicine*#	97	−0.03	96	−0.10	−0.07 (− 0.16; 0.03)	0.165	0.279	0.20

*N* number of children, *CI* confidence interval, *NRS* numerical rating scale, *GPE* global perceived effect (1 = almost gone to 7 = worse than ever)

*change scores from baseline to follow up; **adjusted *p*-value; ***standardized mean difference between groups; # proportion of weeks with medication use

#### Number of days

In both treatment groups, we observed a substantial variation in the change in number of days with a tendency to more substantial improvements in the intervention group (Fig. 2). On average, children reported fewer days with headache after the four-month follow up in both groups compared with baseline, however the mean reduction in number of days with headache was twice as high for children in the intervention group compared with the control group (0.81 vs. 0.41), which was statistically significant (Table 2). The effect size (Cohen’s d) was 0.32. From the responder analysis, illustrated in Fig. 3, it can be seen that about one-third of the children displayed more than a 50% improvement with response being more frequent in the intervention group. The NNT to reach 20% improvement was 7.

#### Intensity

Regarding intensity of headache, we can observe a reduction over time for many children in both groups (Fig. 2). The mean reduction was almost equal between the two groups (0.53 vs. 0.52) and the difference was not statistically significant (Table 2). The effect size (Cohen’s d) was 0.01. In the responder analysis illustrated in Fig. 3, less than a third of the children showed an improvement of at least 20%. The difference between the groups was of negligible magnitude. The NNT to reach 20% improvement was 50.

#### Global perceived effect

As illustrated in Fig. 2, improvement in GPE was more frequent in the intervention group. The mean score was 3.2 in the intervention group and 2.6 in the control group. The difference in mean scoring was significant with a value of 0.61, corresponding to a Cohen’s d of 0.67 (Table 2). When dichotomised, 43.4% reported improvement in the intervention group and 22.0% in the control group, resulting in an odds ratio of 2.8 (95% CI: 1.5–5.3). The number needed to treat was 5.

#### Medicine

As illustrated in Fig. 2, intake of medicine went up or down to a similar degree in both groups, resulting in small reductions on average (Table 2). There was no statistically significant difference detected between groups (0.10 vs. 0.03). Cohen’s d was 0.20. In the responder analysis seen in Fig. 3, both groups showed an almost equal improvement, resulting in an NNT of 40 to reach 20% improvement.

### Secondary outcomes

In the intervention group, 84% of the children reported side effects following at least one consultation and in the control group it was 75%. Side effects reported were mild in nature, most often soreness, headache and fatigue, and they were typically of short duration (0–2 h). There were no serious side effects reported. Details are reported in Additional File 5, Supplementary Table 3.

**Fig. 4:**
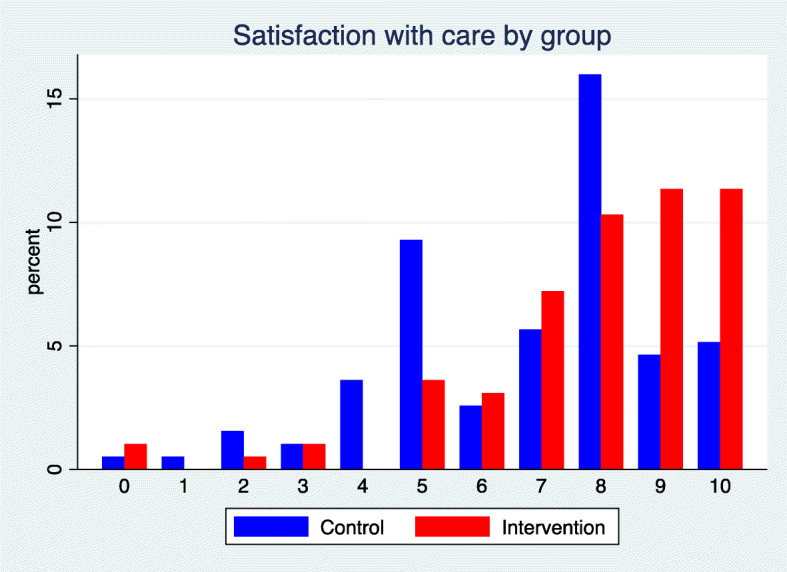
Proportion of children satisfied with participation in the trial as reported by their parents

As seen in Fig. 4, most children were quite satisfied with participation in the trial, but children in the intervention group were on average more satisfied than children in the control group. The difference was statistically significant (*p* < 0.001) with a mean of 7.9 versus 6.8 for the intervention and the control group, respectively. Cohen’s d was 0.49.

### Course of treatment and guessing of treatment group

We intended to expose the children to treatment for 17 weeks and this was achieved in the intervention group, with a median of 16.8 weeks and little variation (10th–90th percentile: 15.7–17.7). The median time was smaller in the control group (15.0) with a more substantial variation (12.3–18.6). There was, however, a distinct difference in the number of consultations, with a median of 11 in the intervention group and 7 in the control group. This reflects a difference in the time scheduling of the consultations. In the control group, we reached the intended gap of 14 days between consultations quite precisely with a median of 14 days, whereas in the intervention group the median was 9 days.

At the four-month follow up, 62% in the intervention group and 59% in the control group guessed the correct randomisation group.

### Sensitivity analyses

Using different definitions of outcomes - as suggested in the initial outcome data analysis report - did not change the results, except for the case of using the original definition for pain intensity. In this case, the results trended towards those observed for number of days with headache, as we expected from our considerations in the initial outcome data analysis (Additional File 5, Supplementary Table 4). The handling of missing values did not seem to affect the results (Additional File 2, Supplementary Tables 5–6). We did not perform analyses of percentiles, as the analysis of mean values gave a clear picture.

## Discussion

### Principal findings

This is the first large-scale randomised clinical trial investigating the effectiveness of chiropractic spinal manipulation in the treatment of recurrent headaches in children. Chiropractic spinal manipulation resulted in significantly fewer days with headaches and better GPE when compared with a validated sham manipulation procedure. There was no difference between groups for pain intensity during headache episodes.

### Previous literature

This positive effect is in line with results seen in studies of adult populations for migraine [36] and cervicogenic headaches [17, 37], whereas the results for tension type headaches are favorable but less conclusive [16, 17]. Also in trials in adults, effects of manual therapy were more pronounced for frequency of headache than for pain intensity [16, 22].

However, the results presented in this article do not distinguish between headache types in the children. Furthermore, it should be noted, that the effect of placebo is possibly larger for children than adults [38], and thus larger effects are needed to demonstrate differences between treatment and sham interventions.

### Strengths and weaknesses

The use of an adaptive design [29–31] allowing for the prioritisation of the primary outcomes based on an interim analysis might be considered unorthodox. However, due to the uncertainty about the measurement properties of the outcomes included, we believed this step to be necessary in order to avoid misjudgments in the priority of outcomes. Actually, the interim analysis led to a down-grading of the outcome medication, which otherwise would probably have been given a higher priority. The interim analysis was pre-planned, blinded for any information about the treatment status of the participant, and performed by an independent statistician, and thus the scientific standard was not compromised. The process was transparent, and the reports are published in the supplementary material.

This study has several strengths. The good compliance and the large sample size resulted in more precise estimates and can facilitate subsequent hypothesis-generating subgroup analyses; utilising weekly SMS text messages as a headache diary reduces recall bias and has been shown to be an efficient and reliable method to collect frequent data [39, 40]; and there has been a structured recording of side effect/adverse event. The blinding appeared to be effective because the observed difference in belief between groups is understandable due to the better outcome in the treatment group likely to have fostered a belief that they received the active treatment [41].

The treatment was individualised with attention to any specific biomechanical dysfunction the child might have had, rather than a standardised treatment given to all. This reflects clinical reality and is likely to make potential recommendations easier to translate into common practice.

We regard having the same, highly experienced chiropractor treating both groups as a way to reach similarity of the clinical encounter across groups, with the only exception being the chiropractic spinal manipulation [42] instead of the sham manipulation. However, we cannot exclude deviations from this optimal scenario due to lack of blinding. Using a highly experienced chiropractor may also question the transferability of results, but using an expertise-based approach can prove to be a strength for the community to subsequently trust and embrace the findings [43]. Future studies must determine the level of expertise needed to obtain similar results.

On average the control group had nearly the intended number of eight consultations over a 16-week period and the intended gap of 14 days between consultations on average, whereas the active treatment group received on average 11 consultations. This is clearly above our expectations for the active treatment group and is due to the fact, that the treatment was pragmatic, i.e. based on the children’s signs and symptoms at each visit, and therefore these could not be precisely planned and did not follow a predefined protocol. On the other hand, the control children followed the predefined pattern of visits to the extent that the parents’ and the clinician’s schedules allowed. It cannot be excluded that the favorable results for patients in the active treatment group are partially due to obtaining more attention - both in quantity and quality. However, the control group actually received an unusually high level of quantitative attention compared to patients not included in this study, such that the effect of additional attention may be limited.

Unfortunately, medication use could not be evaluated in this study due to difficulties interpreting the responses as described in the decision report. Future studies should attempt better ways to include use of medication since the risk of Medication Overuse Headache increases with age [8] and the use of over-the-counter painkillers is worryingly high in adolescence, primarily due to headache [44].

### Implications

Considering the significant consequences of paediatric headache and the lack of effective and safe pharmacological treatment [1, 3, 38], non-pharmacological treatments such as spinal manipulation could be attractive alternatives [45]. The positive results from this study combined with the low risk of adverse events should encourage clinicians and policy-makers to consider spinal manipulation for children with recurrent headaches.

### Unanswered questions and future research

The most important next step is investigating of the long-term effect. One-year follow-up is being completed for the present study and will be reported in a later article. The difference in effect of treatment between GPE (NNT = 3) and frequency (NNT = 7), and the lack of effect on intensity, might imply that frequency and pain intensity alone do not adequately capture ‘improvement’ as experienced by the children. This indicates that there are elements of improvement which are not captured by the investigated outcomes. Before future trials of manipulation or other types of treatment for paediatric headaches are initiated, qualitative studies should investigate further which outcomes are important to children.

Furthermore, considering the complexity of childhood headaches and the large individual differences in response to treatment observed in this study, it is important to identify potential treatment effect modifiers to target treatment efficiently.

## Conclusion

We found that children with recurrent headaches who received chiropractic spinal manipulation experienced fewer days with headaches compared with children receiving sham manipulation. We could not detect a relevant difference in pain intensity between the groups. Children receiving chiropractic spinal manipulation also reported higher self-rated improvement than children receiving sham manipulation. Unfortunately, medication data were unreliable and therefore no conclusions could be drawn on this.

Consequently, since the treatment is easily applicable, of low cost and with no or only mild side effects, chiropractic spinal manipulation might be considered as a valuable treatment option for children with recurrent headache.

## Supplementary Information

**Additional file 1.** The results of the initial outcome data analysis and the resulting decision report.

**Additional file 2.** Tables showing results following imputation of data.

**Additional file 3.** Full reporting check list including page numbers.

**Additional file 4.** Flowchart of SMS-reports.

**Additional file 5.** Additional information in tables.

## Data Availability

Relevant anonymised data are available from the corresponding author on reasonable request.

## Abbreviations

GPE: General Perceived Effect
NNT: Numbers Needed to Treat
NRS: Numerical Rating Scale
SMS: Short Message Service
