# Supplementary material for: Effectiveness of chiropractic manipulation versus sham manipulation for recurrent headaches in children aged 7–14 years - a randomised clinical trial
Source: Chiropr Man Therap. 2021 Jan 7;29:1. doi: 10.1186/s12998-020-00360-3 (PMC7792176; doi:10.1186/s12998-020-00360-3)
Supplement: Supplementary file 1 — Additional file 1. The results of the initial outcome data analysis and the resulting decision report. [file 12998_2020_360_MOESM1_ESM.pdf]

Headache-RCT

Initial outcome data analysis – statistical report

Werner Vach

Background

This is the statistical report about an initial outcome data analysis as part of a randomized controlled trial. The protocol of the trial has been published under the title

Effectiveness of chiropractic manipulation versus sham manipulation
on recurrent headaches in children aged 7-14 years.
Protocol for a randomized clinical trial

(Lynge et al, Chiropractic & Manual Therapies 2019, 27:40 https://doi.org/10.1186/s12998-019-0262-y).

After a first screening for eligibility, the study involved a pre-treatment period of at least 4 weeks, in which parents already answered weekly text messages. At the end of the pre-treatment period, the final decision for inclusion was made as part of an investigation by the primary investigator. The children were then randomized and followed up for 4 months (17 weeks).

The following information on the outcome data have been given in the protocol:

> … the parents and the child together answer the following three questions every Sunday, sent to the parents as a text message (SMS) on their cell phone:
> 1. "How many days has <child's name> had a headache this week? Choose a number between 0 and 7".
> 2. "How will you rate the pain on a scale from 0-10, where 0 is no pain and 10 is the worst pain you can imagine?"
> 3. "How many pills for headache has < child's name> taken this week? 0: none, 1: 1-4, 2: more than 4 pills."
>
> …
>
> At the end of the four months of treatment, all participating families receive a final text message including three questions:
> …
>
> 2. "How has the headache changed since <child's name> started the treatment at the chiropractor? 1. almost gone/disappeared. 2. much better. 3. a little better. 4. same. 5. a little worse. 6. much worse. 7. worse than ever."
>
> …
>
> Primary outcomes
>
> For each week we will consider three variables based on the weekly SMS:
> 1. Number of days with headache per week
> 2. Headache intensity on a pain scale from 0-10 per week
> 3. Number of headache pills per week

> To catch the effect of the intervention, we will consider the average values during the pre-treatment period and the final four weeks of the study period (week 14-17). Three primary outcomes are then given by the change over time, i.e. the difference between the average values from the final four weeks and from the pre-treatment period. The primary outcomes are prioritized as listed above. A fourth primary outcome is the global perceived effect (GPE) after four months based on the final SMS.

The initial outcome data analysis has been motivated and described in the protocol in the following way:

> As mentioned above, our interest is in four potential endpoints, which we have prioritized according to clinical relevance. However, due to lack of experience with using these outcomes in this setting, we do not know whether they are measured in a reliable manner and whether they show a population variation which makes them suitable as an outcome in an RCT. For example, we cannot exclude that there is little variation in some of the intended outcomes across children or that we observe associations with baseline variables which are lower than expected and/or difficult to explain. Such insights may lead to a change in the prioritization of the outcome variables.
>
> We will hence conduct an initial outcome data analysis. With respect to the three SMS questions we will inspect the individual trajectories regarding smoothness and visibility of improvements or deteriorations at the individual level. Next, we will relate the four weeks pre-intervention averages and standard deviations to all baseline characteristics and to each other in order to check whether the observed association patterns can be explained in a reasonable manner. With respect to the change from pre-intervention (four-week averages prior to intervention) to post-intervention (four-week averages at end of trial period) we will consider the joint distribution to judge the existence and magnitude of floor and ceiling effects and to inform the choice between absolute or relative changes. We will also depict the distribution of the change variables themselves, in particular with respect to the degree of variation. Further, we will compare the distribution of the three change variables to judge both the general association as well as specific combination patterns, for example the occurrence of a clear improvement in one question but a stagnation in another question. These analyses will also be stratified by age and gender to judge whether such characteristics may influence the degree and pattern of association. If no clear patterns emerge, the investigation will be extended to a multivariable analysis of all pre- and post-intervention measurements of the three questions. GPE will be included in the analysis of the change variables.
>
> The initial outcome data analysis will be performed by an independent statistician, who will obtain a copy of the data set with baseline characteristics, the weekly results for the three SMS questions and the GPE of the first 100 children. No information on treatment group membership will be provided. The statistician will prepare a statistical report to be distributed among the research team. The research team and the statistician will discuss the results and prepare a further short report summarizing the insights gained and potentially deciding on a reprioritization. We do not use a priori defined criteria for the decision to change the prioritization. In general, many different properties may pop up which may influence the decision. Consequently, it is hard to pre-specify an algorithm. Initial outcome analyses have rarely been performed until now, so there is no established framework for this. However, the decision will be based on a combination of the statistical findings and theory. We will therefore report the entire the process for clarity (statistical report as well as a transparent report of the decision process). All analyses will be blinded and, we can therefore exclude any undue influence on the final

results of the study. Both reports will be finished prior to randomizing the last child in the study and will be included in the supplementary material of the first research publication.

Structure of the report

The report follows the steps outlined in the study protocol. For each step the essential data is visualized in one or several figures. We refer to the figures by name (shown in the lower left corner of each figure), not by number. The figures are collected at the end of the report in the sequence as they appear in the report. In some instances, additional computations are made. The observations made in each step are discussed in each step in the light of the intention of this report to prepare a prioritization of the outcome variables.

Material

The analyses are based on the data oft the first 100 children included in the study. Data on the four outcomes and all variables measured at baseline (i.e. prior to the pre-treatment period) were made available. No information on the actual treatment was provided.

Raw outcome variables used

The following raw variables were available for the analyses:

- The answers to the three weekly SMS questions from week -4 to week 17. Some children were observed for up to 8 weeks prior to start of the study. This data is included, too, and the additional weeks are numbered -5, -6, etc. The variables are
    - Number of days with headache per week
    - Headache intensity on a pain scale from 0 to 10 per week
    - Number of headache pills per week
      (This data was already collected as grouped data with the scores 0: no pills, 1: 1-4 pill, 2; 4 or more pills)

  The variables will appear in the analyses with the abbreviations `days`, `ins` and `med`. They will be verbally rephrased to as "number of days", "intensity" and "number of pills". The numbering of the weeks is from -8 to -1 and then from 1 to 17.

- The global perceived effect asked for in the final SMS at week 17. (Note that the response categories 6 and 7 have not been used by the participants) The variable will appear with the abbreviation `GPE` in the analyses.

Baseline variables used

Since the baseline assessment was performed closely to the start of the pre-treatment period and involved mainly variables to be expected to be stable over time, it is reasonable to compare baseline measurements with measurements from the pre-treatment period.

From the baseline variables available two types of variables were selected. The first are variables for which we wish to see a limited effect on the potential outcomes, as the outcomes should not reflect these child characteristics. Here we selected the variables `age` and `sex`. (According to the protocol, also SES should be included. However, this variable is assessed only at the one-year follow up, and hence it was not available.)

The second group of variables are those for which we expect to see some relation to the weekly measured outcomes at baseline. Here we selected the following variables:

`hduration`	Answer to the question "How long have you suffered from headache? " with the response categories 1: 0.5-1 year; 2: 1-3 years; 3:More than 3 years
`hfreq`	Answer to the question "How often do you have a headache?" with the response categories 1: 1-2 days/week; 2: 3-5 days/week; 3: Almost every day
`hepilen`	Answer to the question "How long does your headache last?" with the response categories 1: Less than 2 hours; 2: From 2 hours-1/2 day; 3: All day: 4: All day and night
`numsymp`	The number of symptoms appearing together with headache and selected from the list "Nausea", "Vomiting", "Dizziness", "Stomach pain", "Visual disturbances", "Spots in front of eyes", "Tingling in arms", "Light sensitivity", "Sound sensitivity", and "Others"
`neckback`	A score based on summing up the questions on the presence of neck pain or back pain last year. 0=neither back nor neck pain; 1= either back or neck pain; 2 = back and neck pain
`freqmed`	The frequency of taking medicine against headache with response categories 1=Never; 2= 1-3 times/month; 3=1-3 times/week; 4=More than 3 times/week (Both prescribed and not-prescribed medicine are considered together, taking the maximal score from both questions. However, only 6 children got prescriptions.)
`htypins`	Intensity of the most typical headache scored on a scale from 0 to 10.
`hsickdays`	Number of sick days last year because of head ache with categories 1=0; 2=1-5/year; 3=5-20/year; 4=More than 20/year;
`gsickdays`	Number of sick days in general over the last years with categories 1=less than 5 days/year; 2=5-20days/year; 3=More than 20 days/year

Predefined outcome variables

According to the protocol, the final analyses should be based on the change in outcomes between the pre-treatment period – assessed by the mean over the final four weeks prior to treatment (i.e. weeks -4, -3, -2, -1) – and the outcome in week 14 to 17 – assessed by the mean in this period. We hence defined the corresponding variables

```
days_4pre, ins_4pre, med_4pre
days_4post, ins_4post, med_4post
days_diff, ins_diff, med_diff
```

It should be noted, that three children have no measurements in the final 4 weeks. However, this was due to a technical problem with the text messages during the start phase of the study. All other children have at least 2 measurements in the final four weeks, and actually for each outcome at least 94 have at least three measurements. No specific measures were applied to take the variation in measurements available into account in the further analyses, i.e. the mean values were just taken across the available measurements. For the four pre treatment weeks measurements were available for all children except of one missing value in one week for `ins` and one child with missing values for `med` in two weeks.

Whenever it turns out to be necessary to define variants of the intended outcome variables, this will be mentioned as an Action.

Statistical methods

For the visual inspection of associations among variables we will use bubble plots, i.e. scatter plots with multiple values shown by circles with a diameter proportional to the number of observations. For quantification we will use the Spearman correlation coefficient. In a few occasions this implies application of the Spearman correlation to a binary variable. In describing the results, the following phrasing was used: $|r|<0.1$: no correlation; $0.1 <|r|<0.2$: slight or weak correlation; $0.2 <|r|<0.4$: moderate correlation; $0.4 <|r|<0.6$: high correlation; ; $0.6 <|r|$: strong correlation.

Further methods used only in a single step are explained at each step. All computations are performed using Stata 15.1.

Step 1: Inspection of individual trajectories

The figures figtrajdays, figtrajins and figtrajmed show the individual trajectories over time for each child. (The pre-period from week -4 to -1 and the post-period from week 14 to 17 are marked by vertical lines.)

We made the following observations:

`days`: The trajectories are rather smooth. There are only few children with a high variation from week to week (e.g. child 43). For some children, we can observe a distinct decrease over time.

`ins`: The trajectories are often rather smooth, but for some children we observe a frequent drop from high intensity to an intensity of 0 (e.g. child 1, 14, 31, 116). A closer look at the data revealed that whenever – with one single exception – no days with pain were reported also an intensity of 0 was reported. (Also the opposite was roughly true: Only at five occasions an intensity of 0 was reported when at least one day with pain was reported.)

Action: A variant of `ins` was defined with the intensity value set to missing whenever no days with pain were reported. This variant is called `ins2`. The corresponding trajectories are shown in figure figtrajins2. We can observe that still some children show a high variation in the weekly intensity. We have also to be aware of that some children have only few weeks with a measured intensity when using this definition.

`med`: Some parents did not follow the instructions to report a 0, 1, or 2, but report higher values, probably the actual number of pills per week. For many children, we never observe values above 2, although the parents chose the value 2 several times during the observation periods. This suggests that some parents followed the instructions, and some did not. (But it might be that all ignored the instructions.) It remains also unclear, whether the parents were consistent over time within a child, as it was allowed that the parents switch in sending the text messages.

Action: On top of the original variable `med`, we define two further variants. In `med2` all values above 2 are recoded according to the original instructions in each child, for whom we observe at least once a value above 2. This variant yields correct values if within a child parents were consistent in either ignoring or not ignoring the instructions and the child took at least once more than 2 pills. In `med3` all reported values above 0 are replaced with a 1, i.e. the information is reduced to the yes/no level with respect to the question whether the child has taken any pill. This variant should yield correct

values independent of the reporting practice of the parents. (We stick in the following to refer to med3 also as “number of pills”, although it reduces the number to the yes/no level.)
With respect to the choice between the three variables we have to balance a potential misclassification in med and med2 versus a loss of information in med3. We will consider in the following all three variants to investigate the impact of the definition on results.
The individual trajectories are shown in Figures figtrajmed2 and figtrajmed3. We can observe typically a high intra-individual variation with weeks without pills and weeks with pills.

The figures figtraj…smooth show smoothed versions of the individual trajectories. We applied in each child the lowess smoother with a bandwidth of 1.2. The children are grouped into groups of 4 to obtain a higher resolution. The grouping is rather arbitrary aiming at having always 4 children differing in the overall level in each group. The colors have no specific meaning and should only help to follow the individual trajectories.
We can observe for all outcomes considered that a rather flat curve or a slowly decreasing curve is a very common pattern, I.e. no substantial change over time. Further we can observe some children with a distinct decrease in the outcome considered, and a few children with a somewhat irregular pattern. These findings are less distinct when considering number of pills.
Overall, these graphs suggest that we can reflect the overall course of a child pretty well by considering the change in mean values between the four weeks prior to treatment and the final four weeks.

Step 2: Association across weekly measurements

To improve our understanding of the role of the three outcomes at the individual level, we take now a look at the associations in the weekly measurements, i.e. we address questions of the type: If a child reports many days with headache, does it also report a high intensity? We have seen in step 1 that the children differ in their overall pain level and that some show a distinct improvement over time. Our interest is here in the association we cannot explain by differences in the overall levels or by a joint improvement over time. Hence we first fit a regression line within each child modelling the outcome as a function of week, and then consider the association of the residuals. We observe the following correlations:

	days	ins	ins2
ins	0.456		
ins2	0.211		
med	0.357	0.462	0.364
med2	0.374	0.490	0.388
med3	0.362	0.500	0.394

The correlation between `days`and `ins2` is rather low. More pronounced correlations can be observed between number of days and number of pills and between intensity and number of pills. So roughly speaking a week with many days with headache or a week with high pain intensity implies also more pain pills in this week, but the association between number of days and intensity at the weekly level is less pronounced.

Step 3: Distribution of pre-treatment outcomes

Action: The definition of `ins2` implies that the number of missing values in both the pre-treatment period and in the final 4 weeks increased. In the pre-treatment period all children have still at least 2 non-missing values, and 82 all 4 measurements. Hence we decided to stick to the original definition. The handling of the final four weeks is discussed later.

Figure fighistpre visualizes the distribution of the pre-treatment outcomes by histograms. (Note that for the medication outcomes each possible value is shown as a separate bar, whereas for the other variables a real grouping is performed.)

We observe that all children reported at least one day with pain in the pre treatment period. However, nearly half of all children reported on average less than 2.5 days, limiting the room for improvement. The intensity variables look here more promising, as many children have values above 4.5 indicating more room for improvement. For the three variables reflecting the number of pills we observe that more than 20% of the children do not report any pills during the pre-treatment period and roughly half of the children score 0 or the next lowest possible value. Since the room for improvement is limited for the majority of children.

Step 4: Relation of pre-treatment outcomes to baseline characteristics

Figures figbaseoutage and figbaseoutsex depict the relation to age and sex. We could observe a slight increase in all outcomes with age. Gender differences could not be observed. Both observations are inline with our expectation of a limited effect of these variables on the outcomes.

Figures figbaseouthduration, figbaseouthfreq, figbaseouthepilen, figbaeoutnumsym, figbaseoutfreqmed and figbaseouthtypins depict the relation to the duration, the frequency and the episode length of headache, to the number of symptoms appearing together with headache, to the frequency of taking medicine against headache and to the intensity of a typical headache. Duration was slightly associated with the number of pills but not with the other outcomes. Frequency was strongly associated with the number of days. It was moderately associated with `ins` but not with `ins2`, which may reflect the difference in the definition, removing the influence of number of days on the intensity. No association could be observed with the number of pills. The length of episodes was not associated with the number of days, and slightly associated with the intensity, in particular with `ins2`, which may again reflect the difference in definition. The relation to the number of pills may be negative, which may reflect that taking pills reduces the episode length. The number of symptoms was not associated with the number of days, but slightly to moderately associated with intensity and number of pills. The frequency of taking medicine was not positively associated with the number of days and with intensity, but highly associated with the number of pills. The intensity of a typical headache was highly associated with intensity, moderately associated with the number of pills, and not positively associated with the number of days.

Figure figbaseoutneckback depicts the association to neck/back pain. No distinct associations could be seen.

Figures figbaseouthsickdays and figbaseoutgsickdays depict the association with the number of sick days because of headache and in general. The number of headache-related sick days was slightly to moderately associated with the intensity and the number of pills, but not with the number of days. A similar pattern was observed for the number of sick days in general.

Overall, we could confirm the validity of the outcomes in the sense of high association with baseline measurements conceptually directly related to the outcome: Frequency of headache was strongly associated with the number of days, the intensity of a typical headache was highly associated with intensity, and frequency of medication was highly associated with the number of pills. For intensity,

we further observed slight to moderate associations with the frequency of headache, the episode length of headache, the number of symptoms appearing together with headache, and the number of sickdays. For number of pills we found slight to moderate associations with the duration of headache, the number of symptoms and the intensity of a typical headache.

A remarkable result was the low association of the number of days with most of the baseline variables.

Step 5: Association among outcomes in the pre-treatment phase

Figure figscatter4pre depicts the associations between the outcomes in the pre-treatment phase. We observe correlations in the magnitude of 0.3-0.4 for the association between intensity and number of pills, a somewhat lower correlation between number of days and intensity (if we use the definition `ins2` reducing the influence of days), and a no correlation between number of days and number of pills.

Step 6: Distribution of outcomes in the final four weeks

Action: With respect to the measurements available for `ins2` in the final four weeks, it turned out that 5 additional children have no measurements, 10 have only 1, and 15 have only 2. We took this into account in the following way: a) ins2_4post was set to 0 in those 5 children who did not report days with pain in these weeks. b) A new variant `ins3_4post` was defined, taking the mean over the last 4 available weeks, but maximally going back to week 11. However, in the 5 children not reporting days with pain the last four weeks the variable was set to 0, and in the 3 with technical problems at the start of the study the variable remained undefined. In the remaining 92 children the mean value stored in `ins3_4post` could be based on 4 measurements in 82 children and in only 1 child only one measurement was available.

Figure fighistpost depicts the distributions of the outcomes in the final four weeks. For number of days we observe a right-skewed distribution with about 15% of the children having less than half a days with headache on average in the final four weeks. For intensity we observe still a rather symmetric distribution with only about 5% of the children with an average intensity below 0.5. For number of pills we observe right skewed distributions with 30% of children reaching a level of 0. So we see here a clear floor effect for number of pills, a slight floor effect for number of days, and nearly no floor effect for intensity.

Step 7: Association of outcomes in the final four weeks

Figure figscatter4post depicts the associations between the outcomes in the final four weeks. We observe a moderate association between number of days and intensity (0.26 and 0.23 when excluding `ins`) and between number of days and number of pills (0.23-0.28), but a more pronounced association between intensity and number of pills (0.40-0.49). (Note: Both associations with the number of pills have increased compared to the pre-treatment phase.)

Step 8: Joint distribution in pre-treatment phase and the final four weeks

Figure figscatterprepost depicts the joint distribution of the outcomes in the pre-treatment phase and the final four weeks. For number of days we can confirm the floor effect already observed when considering the distribution in the four final weeks: Some children seem to reach the 0 level after

starting already with a low number of days. For intensity, we observe no tendency to any floor effect: The few children with a level of 0 at the final week are exactly those 5 with no days with headache in these weeks. The joint distribution of number of pills is dominated by the huge number of children, who are starting with no pills and who are ending with no pills. However, for `med3` we can nevertheless observe a substantial variation in both directions.

Step 9: Distribution of change scores

Figure fighistdiff depicts the distribution of changes scores. For all outcomes we observe a rather symmetric, but slightly left-skewed distribution, indicating the existence of a few children with a distinct improvement. (However, we should remember that the value 3 for GPE refers to "better", not to "equal".)
We can also observe that all change scores except of GPE show a rather distinct mode at 0 or close to 0. Indeed, all distributions expect of `med3diff` have a kurtosis above 3, indicating a more "peaky" distribution than a normal distribution.
All these observations are in line with the original observations made when looking at the individual trajectories: We have a large group of children with nearly no change over time and a few with a distinct improvement.

Figures fighistdiffgender and fighistdiffage show the distribution of the change scores stratified by gender and age. We can observe a tendency towards a more left skewed distribution in girls compared to boys. This holds also for GPE, where we observed a symmetric distribution in girls, but a right skewed distribution in boys. We also observe a higher variation in children above age 11 compared to younger children.

Step 10: Association among change scores

Figure figscatterdiff depicts the association among change scores. We observe slight correlations between the change in number of days and the change in intensity when using `ins2` or `ins3` (<0.2), moderate correlation between the change in number of days and the change in number of pills (0.24-0.34), and slightly more distinct correlations between change in intensity and change in number of pills (0.26-0.41). GPE showed a strong correlation with the change in number of days (r=0.55), a much less distinct correlation with the change in intensity when using `ins2` or `ins3,` (r=0.27 or 0.20, respectively), and also a rather moderate correlation with the change in number of pills (0.24-0-26).

It should be noted that there is only a strong correlation, but a less pronounced agreement between GPE and number of days. In a substantial number of children the GPE was scored as "better" in spite of no improvement in the number of days. We can take a closer look at this in Figure figscatterbyGPE, showing the pairwise joint distributions of the change in number of days, the change in intensity, and the change in number of pills stratified by GPE. We can observe that scoring GPE as "cured, by and large" requires an improvement in number of days as well as at least no deterioration in intensity and number of pills.
The majority of patients scoring "much better" have also an improvement in all three dimensions. An increase in number of pills is sometimes accepted. However, an increase in both number of days and intensity is never accepted. Patients scoring "better" show often only an improvement in number of days or intensity/number of pills, but it still holds that an increase in intensity and number of days is rarely accepted. Patients scoring "the same" are characterized by a change in number of days close to or above 0, and widely varying increases or decreases in the other two dimensions. So this is a sub group of patients for whom the number of days seems to be essential. The few patients scoring "worse" seem to be characterized by a decrease in at least one, but typically at least two

dimensions. Overall, these patterns suggest that the participants vary in balancing number of days versus intensity/number of pills.

The figures figdiffscatteryoung, figdiffscatterold, figdiffscattermale and figdiffscatterfemale depict the association among the change scores when stratified by age or gender, respectively. There are some remarkable differences: The association of `ins3` with the number of pills is much more pronounced in girls (0.42-0.53) than in boys (0.12-0.28). The association of GPE with `ins3` is much more pronounced in boys (0.41) than in girls (0.13) as well as the association of GPE with number of pills is more pronounced in boys (0.30-0.38) than in girls (0.17-0.25). The association of GPE with `ins3` is more pronounced in children above 11 (0.39) than in younger children (r=-0.03). Together with the previously observed differences in the distribution of the change scores when stratifying by age and gender, this may hint to an influence of gender and age on pain perception and scoping strategies. It would be of interest to consider also the analyses presented in figscatterbyGPE stratified by age and gender, but the group sizes are not sufficient for such an analysis.

Step 11: Further statistical considerations

It might be also of interest to take a look at the variation in each change score, as the larger the variation in a change score, the larger might be the possibility to find later a group difference. (This is due to the observation that many children show no variation. Hence this indicates that the natural course of the disease is characterized by no change, and hence any deviation may be due to the intervention.) We hence also took a look at the standard deviation of the change scores. However, as all outcome are measured on different scales, a direct comparison is impossible. We approach this by comparing the observed standard deviation with the maximal possible range of a change. This results in the following values:

	sd	range	sd/range
`days`	1.34	14	0.096
`ins`	1.69	20	0.085
`ins2`	1.82	20	0.091
`med`	0.60	?	
`med2`	0.45	4	0.113
`med3`	0.33	2	0.166
`GPE`	1.00	4	0.249

We can see that after standardization to the possible range, the variations for number of days and intensity are rather comparable, whereas they are increased for the number of pills and for GPE. The latter reflects, however, just that the GPE values cover the whole possible range in contrast to the other variables, and also the conceptual comparability of the different variants of number of pills with number of days and intensity is questionable.

In addition, an observed variation need not necessarily reflect a treatment effect. If may also just reflect inter-individual random differences. We may get indirect insights into this by making use of the fact that we have several pre-treatment measurements in each child, where treatment cannot have any effect and hence we expect rather stable conditions. Within these measurements, we can fit a random intercept/random slope model. The standard deviation of the random slopes gives us information about whether deviations from the assumption of stable conditions may appear within this 4-weeks period, i.e. whether some children increase and other decrease. The standard deviation of the residuals allows us to assess the random variation from week to week. The smaller this

variation, the higher is the chance to detect also small differences over time at the child level. The resulting estimates are shown in the following Table:

	sd(slope)	sd(residual)	range	sd(res.)/range
days	0.24	1.04	7	0.15
ins	0.00	1.81	10	0.18
ins2	0.00	1.46	10	0.15
med	0.00	0.76	?	
med2	0.00	0.49	2	0.25
med3	0.00	0.42	1	0.42

Only for the number of days we could find some evidence for short-term fluctuations. The residual variation was comparable between number of days and intensity when using ins2 (reflecting the decrease in variation intended by this definition.) Number of pills shows a higher residual variation, but this may reflect the binary nature of these variables.

Conclusions and recommendations

Recommendations on the definition of variables

Whereas using the reported number of days as outcome variable is straightforward, there are different ways to use the information on intensity and number of pills.

With respect to intensity, the original definition of the intensity should be discussed. In the original definition (int1) weeks without pain are assigned an intensity of 0. This is a straightforward choice which can be justified in the way that no headache is the lowest possible intensity. However, if we now consider summary scores summing up these values over several weeks, it is questionable whether the resulting score is correctly labelled as “intensity”. We can regard this summary score also as weighted version of a “number of weeks with pain” score, i.e each week with pain is weighted with the intensity in this week. (Similar to the concept of “Quality of Life Adjusted Life Years (QALYs) in health economics”, in which life years are weighted with the quality of life in each year.)
Now it is a crucial point that the summary score for “number of days” is already a weighted version of a “number of weeks with pain” score: Each week with pain is weighted with the number of days with pain. Consequently, the summary scores for “intensity” and “number of days” tend to correlate, in particular as weeks without head ache are rather common. And this correlation is indeed substantial. For the change score we observe a correlation above 0.4. Moreover, in many of our analyses we observe that the results observed for int1 were close to those for number of days.

It might be argued that the decision to use both number of days and intensity as outcomes reflects the wish to regard them as two conceptually independent dimensions of headache. Consequently, the use of int1 might be regarded as suboptimal, and we may want to have an alternative. Such an alternative is also suggested by the empirical observation that many children fluctuate between weeks with no pain days and weeks with high pain intensity. It can be argued that these children experience still a high intensity of headache, even if they have headache only each second week. Consequently, a straightforward idea is to regard the intensity as a missing value in weeks with no pain days. However, this causes some challenges in defining the mean intensity within the final four weeks, as a substantial portion of children have only few weeks with pain in the final four weeks. Hence I recommend allowing to go back further in time in order to be able to include 4 measurements. Details can be found above in the definition of ins3. The “success” of this

alternative can be seen in many analyses, in which the use of `ins2` and `ins3` gave different (and interesting) results compared to the use of `ins1` and could overcome the close resemblance between `ins1` and number of days.

So I recommend using the definition `ins3` instead of `ins1` for the outcome intensity in order to cover two conceptually independent dimensions reflected by outcome variables with low statistical dependence. `ins1` should be considered as a secondary outcome.

With respect to the number of pills, we are confronted with the fact that some parents did not follow the instructions. To address this problem, I considered three different ways to use the recorded information. (See the definition of `med2` and `med3` in Step 1). The variant med3 is conceptually the most convincing one, as it defines a variable not affected by measurement error due to the inconsistency in following the instructions. It turned out, that this variant did not perform differently than the two others (and often performs "best", e.g. it shows the highest correlations with intensity in the analysis of the pre-treatment averages, the averages from the final for weeks, the change scores and when considering the intensity of a typical headache). Hence I recommend using this variant. However, the phrasing should be changed to "medication" to reflect the binary nature of this variable.

General usefulness of the four outcomes

We have no reason to exclude a priori one of the four potential outcomes due to futility. All outcomes have the potential to be able to show a treatment difference between the two groups and can be regarded as valid measurements of the intended constructs.

Likelihood of having a high power to demonstrate treatment effects

The main qualitative difference observed between the outcomes is the tendency to suffer from floor effects. This problem is most pronounced for the number of pills: About a quarter of the children are starting with no pills in the pre-treatment period and hence have no room for improvement. More than half of the children start with less than a half week with a pill and have hence little room for improvement. The problem is less pronounced for number of days, where all children start with at least two days per week. However, also here in some children the room for improvement was limited and we observed some floor effects when looking at the change scores. No floor effects could be observed for intensity.

We also looked at the observed variation of the different outcomes and tried to extract information from the week-to-week variation in the pre-treatment phase. However, this did not give further substantial insights.

Insights into patient relevance

We could observe very distinct differences in the associations of the different outcomes with external variables, indicating differences in the relevance for the patients. Intensity was slightly to moderately correlated with the frequency of headache, the episode length of headache, the number of symptoms appearing together with headache, and the number of sick days. None of these external variables were associated with the number of days. This may suggest that intensity is more relevant for the children than the number of days.
However, when comparing the GPE with the change in outcomes, we observed a strong correlation with the change in number of days, and only a rather moderate correlation with intensity and number of pills. This suggests that a change in the number of days is relevant for the perceived effect. A closer look at the relation between GPE and number of days, intensity and number of pills

together revealed that the participants may highly vary in balancing number of days vs. intensity in their assessment of GPE.

Suggested prioritization

From a statistical point of view, the basic difference between the outcomes is the degree in suffering from lack of room for improvement and floor effects. This suggests giving highest priority to intensity, followed by number of days and finally by number of pills. No recommendation can be made with respect to the placing of GPE in the prioritization.

However, the final decision should take into account also the patient relevance of the different outcomes.

Further recommendations

- Due to the substantial number of children starting with no pills or only one week with a pill, I suggest to plan a further secondary analysis including only children with at least two weeks with a pill.
- There is no reason to use a relative change measure instead of an absolute change measure at the individual level. It would be actually a pure idea to use a relative change. Hence we can stick to the study protocol with respect to the primary analysis.
- The protocol also suggests responder analyses. It is planned to define responders based on the relative change from baseline for the three change scores. I suggest restricting this analysis to the change in intensity, as for number of pills and number of days relative changes are very unstable for a substantial fraction of children starting at low levels.
- Given the distribution of the change scores, it is not unlikely that the intervention is only useful for a small fraction of children, but within these children the effect is remarkable. There is some risk that in this scenario we will observe only a modest difference in the mean change, which will not be statistically significant. I recommend hence performing a sensitivity analysis, based on comparing the mean over the 10%, 6% and 2% percentile of the change scores between the two treatment groups.
- The distributions of and the associations among the outcomes suggest that the perception and scoping strategies with respect to headache and a potential change in headache differ between boys and girls and between older and younger children. This may imply that treatment effects are visible to a varying degree in different subgroups. I hence recommend to plan also sub group analyses with respect to age and gender.

Headache-RCT

Initial outcome data analysis – decision report

Background

This is a report related to an initial outcome data analysis conducted as part of a randomized controlled trial. The protocol of the trial has been published under the title

"Effectiveness of chiropractic manipulation versus sham manipulation
on recurrent headaches in children aged 7-14 years.
Protocol for a randomized clinical trial"

(Lynge et al, Chiropractic & Manual Therapies 2019, 27:40 https://doi.org/10.1186/s12998-019-0262-y). This report summarizes the decisions made based on the results of the initial outcome data analysis, which were reported in a separate report.

Definition of variables

Intensity

The outcome intensity should be represented by the variant `ins2` considered in the statistical report, i.e. the intensity is regarded as missing if no days with pain are reported. We prefer this over for `ins3`, as the latter requires to go back in time to define the post treatment measurements, and this way we may fail to catch an improvement occurring during the last weeks of follow-up. We prefer `ins2` over `ins1`, as we regard it as an advantage to have a variable for intensity, which is not highly correlated with number of days. This will supplement number of days as it reflects the intensity of headache *when present.* However, we cannot exclude that `ins1` or `ins3` are more adequate ways to catch a potential effect. We will analyse these variables as part of sensitivity analyses.

Number of pills

This outcome should be represented by the variant `med3`, i.e. we reduce the information to the yes/no level. This is the only variant with a clear, unambiguous interpretation and the analyses gave no indication of any advantage by using `med1` or `med2`.

General usefulness of the four outcomes

We agreed that all four outcomes considered are useful as primary outcomes.

Prioritization

We regard intensity and number of days as the two most useful outcomes, as they have a clear interpretation and show a distribution promising a rather efficient capture of a

potential effect. We could not see a good argument to prioritize between these two variables, as they both reflect patient-relevant dimensions and we cannot predict, which dimension will be best to capture a potential effect. Consequently, we will rank these two outcomes equally and regard them both as outcomes with highest priority.

The lack of prioritization among these two outcomes with highest priority requires to be taken into account in the planned fixed sequence strategy. We will therefore use a Bonferroni correction for these two outcomes. Consequently, if we reach a p-value of less than 0.025 for at least one of these two outcomes, we will consider formal significance testing of the remaining primary outcomes.

The third primary outcome will be GPE, which we regard as a useful outcome because it is not just a simple summary of the other outcomes. The fourth primary outcome will be the number of pills (`med3`). The low priority of number of pills reflects the uncertainty of the reporting of this, as well as the expected lack of power in showing an improvement in this outcome due to the large number of children starting with no pills.

Further recommendations

- The following sensitivity analyses should be included in the main paper:

    - `ins1` and `ins3` as alternative outcomes
    - Change in number of pills analysed only in children with at least two weeks with a pill in the pre-treatment period
    - Considering the difference in average of the 2%, 6% and 10% percentile of the change scores between the two treatment groups as additional outcome. This should enable us to measure a treatment effect even if only a small group of children is benefiting. Confidence intervals and p-values will be based on a non-parametric bootstrap.

- Responder analyses should be performed as suggested in the original protocol. We consider adherence to the original protocol as more important than the potential weaknesses of the analyses.

- Age and gender should be included in subgroup analyses. However, subgroup analyses will be not part of the first, main paper, but of a secondary paper.

August 30, 2019

Werner Vach
Henrik Wulff Christensen
Kristina Boe Dissing
Lise Hestbæk
Susanne Lynge
