# Supplementary material for: Effectiveness of chiropractic manipulation versus sham manipulation for recurrent headaches in children aged 7–14 years - a randomised clinical trial
Source: Chiropr Man Therap. 2021 Jan 7;29:1. doi: 10.1186/s12998-020-00360-3 (PMC7792176; doi:10.1186/s12998-020-00360-3)
Supplement: Supplementary file 2 — Additional file 2. Tables showing results following imputation of data. [file 12998_2020_360_MOESM2_ESM.pdf]

Additional file 2. Multiple imputation.

We used multiple imputation to perform sensitivity analyses with respect to handling missing values. The imputation was applied to the data on the weekly SMS-messages including all 199 children randomised. Consequently, the data set included for each of the three outcomes *number of days with headache*, *pain intensity* and *intake of pain medication* the four pre-treatment responses as well as the 17 responses from each week after start of treatment. In addition, it included the outcome variable GPE. Age, sex and intervention were included as additional variables completely observed for all children.

Multiple imputations were based on chained equations. The prediction equations for the response to one outcome in a single week included the responses of the previous three weeks and of the following three weeks for the same outcome, the responses to the other two outcomes for the same week, the GPE and age, sex and the intervention. The prediction equation for GPE included the average pre-treatment values for the threee outcomes as well as the averages over the final four weeks.

For all outcomes including GPE we used an ordinal logistic regression model and allowed augmentation in the case of perfect prediction. All covariates were entered as continuous covariates, adding also the square of age. With respect to taking the intervention into account, we considered two variants: In the first we include the intervention as a covariate in all prediction equations. In the second we performed multiple imputation separately in the two intervention groups.

One additional challenge arose from a structural relationship between the three responses and the specific definition of the pain intensity score excluding weeks with no days on pain. If the number of days with pain in a week is 0, children (nearly) always reported to take no medication and the pain intensity has no influence on the final results. We tackled this challenge by the following three measures: 1) Prediction of the pain intensity and medication was restricted to the case that the number of days was larger than 0. 2) When using pain intensity or medication from one week to make predictions for another week, we added an indicator whether the number of days was equal to 0 and set the predictor to 0, such that either the information of no days with headache or the observed values for pain intensity or medication, respectively, were used for prediction. 3) In predicting the number of days, we used the intensity and medication values of the previous week instead of the current week.

We used 20 imputations after a burn in phase of 10 imputations. The imputed data sets were analysed using the same methods as in the complete case analysis.

Supplementary Table 5. Results on the four primary outcomes based on multiple imputation (variant 1)

	**Control**		**Intervention**					
**Outcome**	N	Mean	N	Mean	Difference in mean (95% CI)	p-value	p-value**	Cohen's d
Number of days*		-0.41		-0.84	-0.43 (-0.80;-0.06)	0.021	0.015	0.33
Intensity (NRS) *		-0.56		-0.54	0.03 (-0.44; 0.49)	0.914	0.970	0.02
GPE		3.24		2.64	-0.61 (-0.87;-0.34)	<0.001	<0.001	0.64
Medicine*#		-0.04		-0.11	-0.07 (-0.17; 0.03)	0.150	0.272	0.21

N: number of children; CI: confidence interval; ES: effect size; *: change scores from baseline to follow-up **: adjusted p-value; # fraction of weeks with medicine; NRS: numerical rating scale; GPE: global perceived effect (1= almost gone to 7 = worse than ever)

Supplementary Table 6 Results on the four primary outcomes based on multiple imputation (variant 2)

	**Control**		**Intervention**					
**Outcome**	N	Mean	N	Mean	Difference in mean (95% CI)	p-value	p-value**	Cohen's d
Number of days*		-0.39		-0.84	-0.44 (-0.82;-0.07)	0.020	0.014	0.34
Intensity (NRS) *		-0.57		-0.53	0.04 (-0.42; 0.49)	0. 878	0.978	0.02
GPE		3.24		2.64	-0.60 (-0.86;-0.33)	<0.001	<0.001	0.64
Medicine*#		-0.07		-0.11	-0.05 (-0.13; 0.04)	0.138	0.268	0.22

N: number of children; CI: confidence interval; ES: effect size; *: change scores from baseline to follow-up **: adjusted p-value; # fraction of weeks with medicine; NRS: numerical rating scale; GPE: global perceived effect (1= almost gone to 7 = worse than ever)
