# Supplementary figures and images for: Effectiveness of chiropractic manipulation versus sham manipulation for recurrent headaches in children aged 7–14 years - a randomised clinical trial
Source: Chiropr Man Therap. 2021 Jan 7;29:1. doi: 10.1186/s12998-020-00360-3 (PMC7792176; doi:10.1186/s12998-020-00360-3)

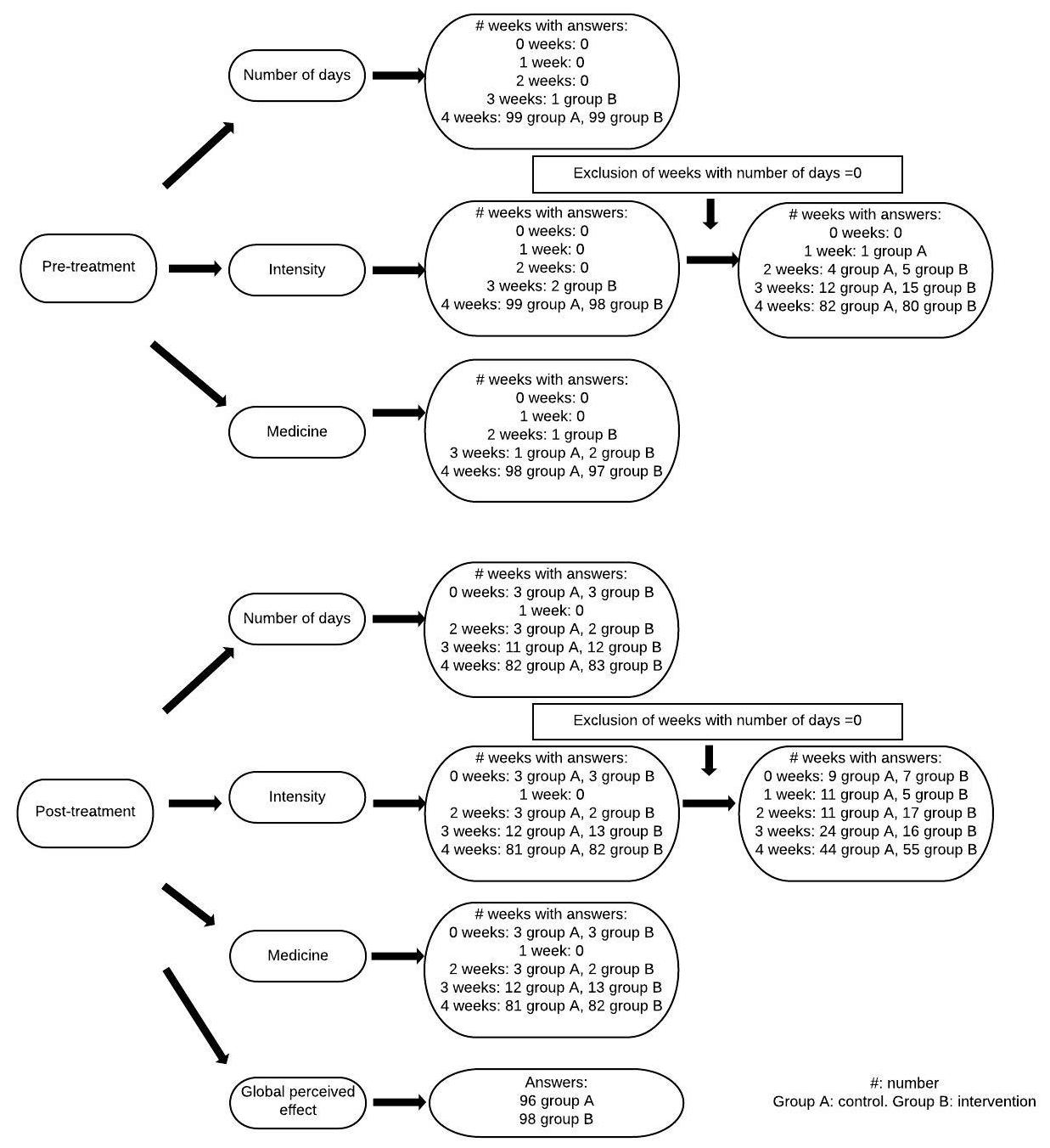

Supplement: Supplementary file 4 — Additional file 4. Flowchart of SMS-reports. [file 12998_2020_360_MOESM4_ESM.jpg]
