# Supplementary material for: Effectiveness of chiropractic manipulation versus sham manipulation for recurrent headaches in children aged 7–14 years - a randomised clinical trial
Source: Chiropr Man Therap. 2021 Jan 7;29:1. doi: 10.1186/s12998-020-00360-3 (PMC7792176; doi:10.1186/s12998-020-00360-3)
Supplement: Supplementary file 5 — Additional file 5. Additional information in tables. [file 12998_2020_360_MOESM5_ESM.pdf]

Additional File 5, supplementary tables

Supplementary Table 1. Baseline characteristics of the participants

	**Intervention group (N=99)**	**Control group (N=100)**
Sex, boy, no (%)	45 (45%)	40 (40%)
Age at inclusion (mean, SD)	10.9 (2.1)	10.7 (2.0)
**Medicine use**		
Non-prescriptive (yes)	92 (93%)	88 (88%)
Prescriptive medicine (yes)	5 (5%)	3 (3%)
**Previous examination* (yes)**	63 (64%)	54 (54%)
Examination by general practitioner	58 (59%)	52 (52%)
**Previous treatment* (yes)**	18 (18%)	23 (23%)
Treatment by chiropractor	8 (8%)	10 (10%)
**Previous trauma head and/or neck (yes)**	78 (79%)	73 (73%)
**Reasons for sick leave***		
Headache	84 (85%)	79 (79%)
**Sick days latest year due to headache**		
0 days	13 (13%)	15 (15%)
1-5 days	45 (46%)	46 (46%)
5-20 days	36 (36%)	34 (34%)
More than 20 days	5 (5%)	5 (5%)
**Neck pain within latest year (yes)**	57 (58%)	70 (70%)
**Back pain within latest year (yes)**	43 (43%)	44 (44%)
**Parents and/or sibling with headache (yes)**	73 (74%)	75 (75%)
**Allergies (yes)**	30 (30%)	37 (37%)
**Frequency of suffering from stomachache**		
No	20 (20%)	14 (14%)
Sometimes (1-12 times per year)	47 (48%)	53 (53%)
Often (more than 1 time per month)	32 (32%)	33 (33%)
**Number of weekly sports sessions**		

0 times	5 (5%)	6 (6%)
1-3 times	72 (73%)	68 (68%)
More than 3 times	22 (22%)	26 (26%)
**Number of screen time hours per day**		
0-1 hour	2 (2%)	14 (14%)
2-4 hours	75 (76%)	64 (64%)
More than 4 hours	22 (22%)	22 (22%)
**Numbers of hours of sleep per night**		
6-8 hours	21 (21%)	18 (18%)
9-12 hours	78 (79%)	82 (82%)

* For these variables the frequency of the most frequent category is reported

Supplementary Table 2. Headache characteristics at baseline

	Control (n=100)	Intervention (n=99)
	n (%)	n (%)
**Duration**		
0.5 to 1 year	22 (22%)	17 (17%)
1 to 3 years	53 (53%)	47 (47%)
More than 3 years	25 (25%)	35 (35%)
**Frequency**		
1-2 days a week	40 (40%)	38 (38%)
3-5 days a week	46 (46%)	49 (49%)
Almost every day	14 (14%)	12 (12%)
**Duration of attack**		
Less than 2 hours	13 (13%)	12 (12%)
2 hours up to half a day	67 (67%)	64 (65%)
The whole day	19 (19%)	20 (20%)
All day and all night	2 (2%)	2 (2%)
**Typical onset of attack**		
Morning	10 (10%)	11 (11)
Before noon	17 (17%)	21 (21%)
Afternoon	27 (27%)	19 (19%)
Evening/night	4 (4%)	0 (0%)
Varies over day and night	42 (42%)	48 (48%)
**Typical onset place**		
The entire head	15 (15%)	13 (13%)
Back side of the head	4 (4%)	3 (3%)
One side of the head	13 (13%)	8 (8%)
The neck	2 (2%)	2 (2%)
The forehead	32 (32%)	38 (38%)
Behind one eye	6 (6%)	5 (5%)
Varies a lot	28 (28%)	30 (30%)
**Other cooccurring symptoms**		
Nausea	56 (56%)	51 (52%)
Vomiting	22 (22%)	21 (21%)
Dizziness	53 (53%)	53 (54%)
Stomachache	37 (37%)	31 (31%)
Visual disorder	22 (22%)	29 (29%)
Seeing dots	17 (17%)	24 (24%)
Numbness arms	8 (8%)	7 (7%)
Light sensitivity	48 (48%)	61 (62%)
Sound sensitivity	57 (57%)	65 (66%)
Other symptoms	9 (9%)	11 (11%)
**Activities/pain causing headache (yes)**		
Neck pain	53 (53%)	42 (42%)

Back pain	24 (24%)	19 (19%)
Stress	43 (43%)	36 (36%)
Sitting	34 (34%)	41 (41%)
Reading	40 (40%)	34 (34%)
Sports	29 (29%)	27 (27%)
Computer/tv	51 (51%)	50 (51%)
Menstruation	2 (2%)	8 (8%)
**Activities easing headache (yes)**		
Lying down	51 (51%)	59 (60%)
Sleeping	82 (82%)	83 (84%)
Eating	31 (31%)	29 (29%)
Drinking	53 (53%)	55 (56%)
Fresh air	44 (44%)	50 (51%)
Sports	13 (13%)	15 (15%)
Medicine	74 (74%)	76 (77%)
**Primary outcome values at baseline**	Mean (p10-p90	Mean (p10-p90)
Number of days with headache	2.8 (2.5-3.1)	2.7 (2.5-3.0)
Intensity of headache (NRS)	5.2 (5.0-5.5)	5.2 (4.9-5.4)
Proportion of weeks with medicine	0.4 (0.4-0.5)	0.5 (0.4-0.5)

Supplementary Table 3. Reported reactions to treatment by group by total number of consultations

	Number of children with reaction, n (%)*		Number of consultations with reaction, n (%)		Number of consultations with reaction by child, range	
	Control (N = 98)	Intervention (N = 96)	Control (N = 664)	Intervention (N = 1052)	Control	Intervention
Fatigue (total)**	35 (35.7%)	25 (26.0%)	69 (10.4%)	80 (7.6%)	0-5	0-11
0-2 hours	30 (30.6%)	20 (20.8%)	59 (8.9%)	46 (4.4%)	0-5	0-8
2-24 hours	7 (7.1%)	11 (11.5%)	7 (1.1%)	20 (1.9%)	0-1	0-6
24-48 hours	3 (3.1%)	6 (6.3%)	3 (0.5%)	14 (1.3%)	0-1	0-5
Headache (total)**	36 (36.7%)	50 (52.1%)	53 (8.0%)	108 (10.3%)	0-5	0-12
0-2 hours	23 (23.5%)	35 (36.5%)	34 (5.1%)	69 (6.6%)	0-5	0-11
2-24 hours	14 (14.3%)	20 (20.8%)	14 (2.1%)	26 (2.5%)	0-1	0-3
24-48 hours	5 (5.1%)	9 (9.4%)	5 (0.8%)	11 (1.1%)	0-1	0-2
Persistent	0 (0%)	2 (2.1%)	0 (0%)	2 (0.2%)	0	0-1
Local soreness (total)**	37 (37.8%)	50 (52.1%)	61 (9.2%)	139 (13.2%)	0-4	0-9
0-2 hours	25 (25.5%)	38 (39.6%)	38 (5.7%)	73 (6.9%)	0-4	0-9
2-24 hours	15 (15.3%)	27 (28.1%)	16 (2.4%)	43 (4.1%)	0-2	0-5
24-48 hours	5 (5.1%)	12 (12.5%)	6 (1.0%)	21 (2.0%)	0-2	0-4
Persistent	1 (1.0%)	2 (2.1%)	1 (0.2%)	2 (0.2%)	0-1	0-1
Nausea (total)**	7 (7.1%)	9 (9.4%)	11 (1.7%)	13 (1.2%)	0-3	0-4
0-2 hours	5 (5.1%)	5 (5.2%)	7 (1.1%)	8 (0.8%)	0-2	0-4
2-24 hours	4 (4.1%)	0 (0%)	4 (0.6%)	0 (0%)	0-1	0
24-48 hours	0 (0%)	5 (5.2%)	0 (0%)	5 (0.5%)	0	0-1

Stomach ache (total)**	3 (3.1%)	4 (4.2%)	4 (0.6%)	5 (0.5%)	0-2	0-2
0-2 hours	3 (3.0%)	0 (0%)	4 (0.6%)	0 (0%)	0-2	0
2-24 hours	0 (0%)	2 (2.1%)	0 (0%)	2 (0.2%)	0	0-1
24-48 hours	0 (0%)	3 (3.1%)	0 (0%)	3 (0.3%)	0	0-1
Vomit (total)**	1 (1.0%)	1 (1.0%)	1 (0.2%)	1 (0.1%)	0-1	0-1
0-2 hours	0 (0%)	0 (0%)	0 (0%)	0 (0%)	0	0
2-24 hours	1 (1.0%)	1 (1.0%)	1 (0.2%)	1 (0.1%)	0-1	0-1
24-48 hours	0 (0%)	0 (0%)	0 (0%)	0 (0%)	0	0
Appearance of visual and/or sensibility disorder						
Yes	0 (0%)	0 (0%)	0 (0%)	0 (0%)	0	0

*A child can appear in multiple subcategories within the same type of reaction, since they could have different degrees of reaction at different consultations.

**Number of children or consultations with at least one reaction of that type.

Supplementary Table 4. Results based on alternative definitions of pain intensity and medication use as described in the initial outcome data analysis report

	**Control**		**Intervention**					
	N	Mean	N	Mean	Difference in mean (95% CI)	p-value	p-value*	Cohen's d
Change in intensity (int1) (NRS 0-10)*	97	-1.04	96	-1.23	-0.18 (0.32; -0.69)	0.476	0.367	0.10
Change in intensity (int3) (NRS 0-10)**	91	-0.94	88	-0.85	0.09 (0.64; -0.46)	0.749	0.714	0.05
Change in fraction of weeks with medicine***	53	-0.19	55	-0.17	0.03 (0.15; -0.10)	0.673	0.565	0.08

*Original definition: weeks without pain are assigned an intensity of 0

** The mean over the last available four weeks with headache, but maximally going back 7 weeks

***Analysed only in children with at least two weeks with a pill in the pre-treatment period
